# Meta-analysis of duloxetine vs. pregabalin and gabapentin in the treatment of diabetic peripheral neuropathic pain

**DOI:** 10.1186/1471-2377-9-6

**Published:** 2009-02-10

**Authors:** Sibilia Quilici, Jeremy Chancellor, Mickael Löthgren, Dominique Simon, Gérard Said, Trong Kim Le, Ana Garcia-Cebrian, Brigitta Monz

**Affiliations:** 1 Health Economics & Outcomes Research, i3 Innovus, Uxbridge, UK; 2 Health Economics & Outcomes Research, i3 Innovus, Uxbridge, UK; 3 Innovus Research (UK) Ltd, High Wycombe, UK; 4 Service de Diabétologie, Hôpital de la Pitié, Paris, France; 5 Service de Neurologie, Hôpital le Kremlin Bicêtre, Paris, France; 6 Global Health Outcomes, Eli Lilly and Company, Indianapolis, IN, USA; 7 European Health Outcomes, Eli Lilly and Company Limited, Windlesham, Surrey, UK; 8 Global Health Economics & Outcomes Research, Boehringer Ingelheim GmbH, Ingelheim, Germany

## Abstract

**Background:**

Few direct head-to-head comparisons have been conducted between drugs for the treatment of diabetic peripheral neuropathic pain (DPNP). Approved or recommended drugs in this indication include duloxetine (DLX), pregabalin (PGB), gabapentin (GBP) and amitriptyline (AMT). We conducted an indirect meta-analysis to compare the efficacy and tolerability of DLX with PGB and GBP in DPNP, using placebo as a common comparator.

**Methods:**

We searched PubMed, EMBASE, CENTRAL databases and regulatory websites for randomized, double-blind, placebo-controlled, parallel group or crossover clinical trials (RCTs) assessing DLX, PGB, GBP and AMT in DPNP. Study arms using approved dosages with assessments after 5–13 weeks were eligible. Efficacy criteria were: reduction in 24-hour pain severity (24 h PS) for all three drugs, and response rate (≥ 50% pain reduction) and Patient Global Impression of Improvement/Change (PGI-I/C) for DLX and PGB only. Tolerability criteria included: discontinuation, diarrhoea, dizziness, headache, nausea and somnolence. Direct comparisons versus placebo were conducted with pooled fixed – and random-effects analyses on endpoints reported in at least two studies of each drug. Indirect comparisons were performed between DLX and each of PGB and GBP using Bayesian simulation.

**Results:**

Three studies of DLX, six of PGB, two of GBP and none of AMT met the inclusion criteria. In random-effects and fixed-effects analyses of DLX, PGB and GBP, all were superior to placebo for all efficacy parameters, with some tolerability trade-offs. Indirect comparison of DLX with PGB found no differences in 24 h PS, but significant differences in PGI-I/C, favouring PGB, and in dizziness, favouring DLX were apparent. Comparing DLX and GBP, there were no statistically significant differences.

**Conclusion:**

From the few available studies suitable for indirect comparison, DLX shows comparable efficacy and tolerability to GBP and PGB in DPNP. Duloxetine provides an important treatment option for this disabling condition.

## Background

Neuropathic pain is often associated with diabetic peripheral neuropathy and is defined as pain initiated or caused by a primary lesion or dysfunction in the nervous system [1]. In a recent cross-sectional study in the UK, the overall prevalence of chronic (>1 year) painful peripheral neuropathy was estimated to be 16.2% among patients with diabetes compared with 4.9% among matched controls [2]. The rising prevalence of type 2 diabetes is likely to increase the burden of diabetic peripheral neuropathic pain (DPNP) [3].

The main symptoms of DPNP are burning or shooting pain in the lower limbs and feet, usually occurring for more than three months. Currently, there are no approved treatments that restore nerve function. A major goal of pharmacological treatment in DPNP is therefore to control pain. Simple analgesics may provide partial, short-term relief, but more specifically targeted drugs are normally required for sustained control of pain of neuropathic origin.

Amitriptyline, a tricyclic antidepressant (TCA) first marketed in the 1960s, is not licensed for treatment of DPNP. However, along with another TCA (nortriptyline), it is recommended in the British National Formulary as a drug of choice for treating DPNP. More recently, the use of anticonvulsants has been proposed for the treatment of neuropathic pain. Gabapentin is licensed for the treatment of neuropathic pain in Europe and for the treatment of post-herpetic neuralgia, a specific type of neuropathic pain, in the US. Pregabalin was approved in 2004 for the treatment of peripheral neuropathic pain in Europe, and in 2005 for the treatment of neuropathic pain associated with diabetic peripheral neuropathy and postherpetic neuralgia (PHN) in the US. Duloxetine is a relatively balanced and potent reuptake inhibitor of serotonin and norepinephrine, approved in Europe and the US for the treatment of DPNP. It was first approved as an antidepressant for the treatment of major depressive disorder (MDD).

The aims of the meta-analysis were twofold. The first was to summarize the efficacy and tolerability of drug treatments licensed or recommended for DPNP by statistically pooling the available data from randomized, placebo-controlled trials. The second aim was to compare the efficacy and tolerability of duloxetine with pregabalin, gabapentin and amitriptyline. As most of the controlled clinical trials of these drugs are comparisons with placebo and very few head-to-head comparisons exist, an indirect approach was chosen using placebo as a common comparator.

## Methods

### Sources

A comprehensive and systematic search of the published literature for trials of duloxetine (DLX), pregabalin (PGB), gabapentin (GBP) and amitriptyline (AMT) in the treatment of DPNP was performed during January 2005 using PubMed, EMBASE and CENTRAL databases. The search strategy was not limited by year or language of publication. Internal study reports of all trials of duloxetine in the treatment of DPNP were provided by the primary study sponsor, Eli Lilly and Company. We are not aware of any trials of duloxetine conducted since the date of search. In addition, the Food and Drug Administration (FDA) and European Medicines Agency (EMEA) websites were searched for available reviews of PGB and GBP. As PGB had only recently been licensed for DPNP, it was considered that all trials completed by the manufacturer would be identified through these websites.

### Study selection and validity assessment

The drugs considered for inclusion in the meta-analysis were those licensed (DLX, PGB, GBP) or recommended (AMT) for DPNP, subject to a minimum requirement for two eligible studies of any drug. Identified references were screened using title, abstract and keywords. Studies were considered potentially eligible for inclusion in the meta-analyses of each individual drug if they were randomised, double-blind, placebo-controlled trials in diabetic neuropathy or diabetic peripheral neuropathic pain with a treatment duration of 5–13 weeks, or longer provided that results were reported for this duration. Studies could be of parallel group or crossover design, but crossover studies had to demonstrate sufficient washout period, randomisation of the order of study treatment and that subjects had stable disease over the study period. Evidence of sample size calculations for the primary efficacy variable was required, consistent with CONSORT recommendations for the reporting of clinical trials, as adopted by BioMed Central[4] and other journals. Eligibility was confirmed on review of full publications and/or study reports against the above criteria.

### Data abstraction

Study design data including design synopsis, treatment comparators, dosage, titration schedule and duration of treatment were abstracted, along with baseline characteristics including summary statistics of pain severity, age and sex. Summary efficacy and tolerability outcomes were also abstracted. Data were entered into spreadsheets by one author (ML) and were verified by another (SQ).

### Selection of outcome measures

By assessing the commonality of outcome measures available across drugs and across the eligible studies of each drug, a set of outcomes was identified for which sufficient data were available for pooling, notwithstanding minor differences in reporting.

The primary assessment of treatment efficacy, available for all drugs, was 24-hour average pain severity (24 h PS), treatment response and overall health improvement. 24 h PS was recorded by patients in daily or weekly diaries on an 11-point ordinal scale, ranging from 0 = no pain to 10 = worst pain possible. Treatment efficacy outcomes only partially available were treatment response and patient global impression. Treatment response was defined as at least a 50% reduction in 24 h PS score from baseline, and was reported as proportions of responders. The overall health improvement was measured on the Patient Global Impression of Improvement/Change (PGI-I/C) questionnaire, a 7-point ordinal, categorical scale describing patients' reported impressions ranging from "very much improved" to "very much worse" [5].

The most frequently reported tolerability outcomes were considered. These included premature discontinuation due to lack of efficacy and due to adverse events (AEs), as well as the AE symptoms reported most frequently (>5%) in patients receiving DLX and corresponding with those reported in the studies of PGB and GBP: diarrhoea, dizziness, headache, somnolence and nausea. By definition, only symptoms reported in common between drugs could be included in the indirect meta-analyses. Treatment effects for continuous variables, such as 24 h PS, were estimated as the absolute difference between the mean change from baseline to study endpoint. Treatment effects for discrete variables (PGI-I/C, response rates and AE incidence rates) were estimated as log-odds ratios.

To test for the significance of between-study differences, analysis of variance (ANOVA) tests were used for continuous baseline measures (age, treatment effect) and the Cochrane-Mantel-Haentzel test was used for sex.

### Quantitative data synthesis

Direct meta-analyses were performed to estimate effect sizes for each drug compared to placebo, using classical frequentist fixed-effects (FE) and random-effects (RE) models. Studies were pooled by weighting the treatment differences by their inverse variances. Comparisons between drug and placebo were expressed as mean differences (*θ*) with 95% confidence intervals. Treatment differences between studies were tested using the Mann-Whitney U-test. FE and RE models were estimated and Cochrane Q-tests, I-squared and *τ*^2 ^statistics were used to test for and quantify between-study heterogeneity. Forest plots were generated to assess the extent of this visually. Superiority tests were performed for the direct comparisons of each active drug with placebo for each outcome, using a one-sided 95% confidence interval (CI). The number needed to treat (NNT) – the number of patients that need to be treated with drug compared to placebo to obtain one additional responder – and the number needed to harm (NNH) for discontinuation due to AEs were calculated. When 95% CIs for NNTs and NNHs were not significant (e.g. if they included a negative CI bound) the sign ∞ was used for the undefined bound [6]. The NNTs/NNHs were derived from the estimated treatment differences (*θ*) and from event probabilities in the control group [7].

Due to the absence of head-to-head trials, the comparisons of DLX with the other active treatments were made by means of indirect meta-analyses, using placebo as a common comparator. These were performed using a Bayesian simulation approach. The adjusted outcomes of active treatments vs. placebo from the direct comparison were pooled across available studies, to derive estimates of *δ*, the mean difference in treatment effect between DLX and each comparator, along with 95% credibility intervals. Non-informative prior distributions of *θ *and the between-study heterogeneity (*τ*^2^) were used.

Non-inferiority tests were performed for the comparisons of 24 h PS between DLX and the other drugs. A difference of 2 points on the 11-point scale was selected as the non-inferiority margin, based on previous research into the correspondence of 24 h PS scores with "very much improved " and "much improved" ratings on the PGI scale [8]. For the remaining outcomes, for which no consensus exists on the minimum clinically meaningful difference, superiority tests were performed.

## Results

### Literature search

**Figure 1:**
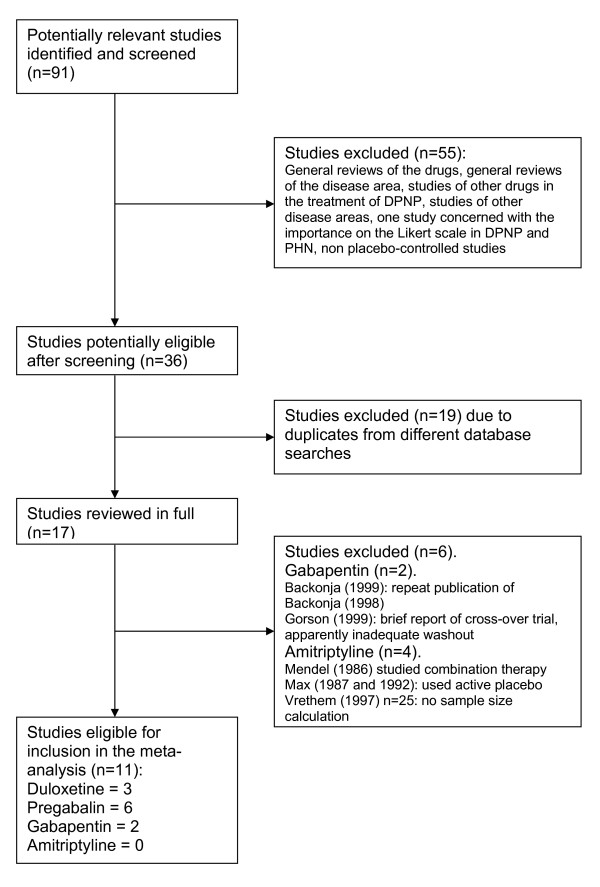
**Flow diagram of systematic review to identify eligible studies**.

Figure 1 presents the results of the literature search, screening and review for those studies deemed eligible for inclusion in the meta-analysis. From 91 potentially relevant publications, 11 were included in the meta-analysis. Of the four randomized studies of AMT potentially eligible, one had a very small sample (n = 25) with no sample size calculation and was, therefore, excluded[9] Two studies included benzotropine as an active placebo (to preserve blinding by mimicking the anticholinergic side-effects of amitriptyline). This precluded any tolerability comparison with no treatment. Of these two studies, one was a crossover study with no washout period. In the other, AMT was not directly compared to placebo. The sole remaining eligible study involving AMT was one of the studies of PGB (DPN-040), which included AMT as a control in addition to placebo. Due to the pre-specified minimum requirement for two eligible studies of any drug, AMT was eliminated from the meta-analysis. Hence, the meta-analysis included DLX (three unpublished studies at the time of searching, available as manufacturer's study reports)[10,11], GBP (two published studies)[12,13] and PGB (six studies, comprising four unpublished at the time of searching, available as EMEA Scientific Discussions, and two published studies) [14-16]. The three DLX studies included 679 patients on active treatment and 339 on placebo; the GBP studies included 114 patients on active treatment and 111 on placebo and the PGB studies included 988 patients on active treatment and 478 on placebo.

For the DLX vs. PGB comparison, three efficacy outcomes were considered: 24 h PS, pain response and PGI-I/PGI-C, and seven tolerability outcomes: premature discontinuation due to adverse events, due to lack of efficacy and due to other reasons, dizziness, somnolence, headache and diarrhoea. For the DLX vs. GBP comparison, only the 24 h PS efficacy comparison was possible due to lack of GBP data. However, all eight tolerability outcomes were possible, comprising the seven described above plus nausea.

Some outcome measures were excluded as they were reported for some but not all of the drugs, or for some but not all trials of a drug. The three studies of DLX included some efficacy outcomes not present in the other drug studies. These included the night pain severity, the Brief Pain Index (BPI), the Clinical Global Impression of Severity (CGI-S), all of which were absent from studies of the other drugs, and the Short Form McGill Pain Questionnaire (SF-MPQ), which was included in two of the six PGB studies and one of the two GBP trials. Because it is an antidepressant, the studies of DLX in DPNP excluded patients with diagnosed depression to avoid biasing estimates of the direct effect of DLX on DPNP. This restriction did not apply to GBP and PGB.

#### Study characteristics

Table 1 illustrates the design and baseline characteristics of the studies included in the meta-analyses. The treatment duration varied between 5 and 12 weeks and the study dosage of individual drugs varied. Only study arms using therapeutic dosage corresponding to regulatory labelling were eligible for inclusion. In testing for overall between-study heterogeneity, significant differences were detected in the proportions of patients by sex (p < 0.0001) and by co-morbid type 2 diabetes (p < 0.0001), while no significant differences were found in baseline pain severity or age.

**Table 1:** Study design and patient baseline characteristics.*

**Study**	**Drug^a^**	**Dose**	**Titration**	**Randomised** **patients **(N)	**Treatment** **duration ** (weeks)	**Baseline severity** **(24-hour** **pain intensity** **score) Mean (sd)**	**Diabetes** **type** (% Type 2)	**Diabetes** **Duration** (years) Mean (sd)	**Diabetes** **Duration** (years) Median	**DPNP** **Duration** (years) Mean	**Age** (years) Mean (sd)	**Gender **(% male)
***Duloxetine***												
HMAW	Duloxetine	60 mg QD		112	12	6.01 (1.69)	87.7	11.42 (8.20)	9.87	3.81	59.21 (11.61)	69.3
	Duloxetine	60 mg BID	3 days	109		5.85 (1.38)	90.3	10.06 (8.95)	7.49	3.45	60.5 (10.84)	60.2
	Placebo			115		5.73 (1.52)	90.4	11.44 (11.26)	7.52	4.03	60.42 (10.5)	51.3

HMAVa	Duloxetine	60 mg QD		114	12	6.12 (1.62)	91.2	9.74 (9.55)	6.35	3.59	59.71 (11.17)	64.9
	Duloxetine	60 mg BID	3 days	112		6.21 (1.54)	92	9.88 (10.01)	6.65	4.38	61.46 (9.94)	54.5
	Placebo			108		5.85 (1.42)	89.8	11.08 (9.09)	9.11	3.53	60.81 (10.57)	63.9

HMAVb	Duloxetine	60 mg QD		116	12	5.55 (1.12)	80.2	14.64 (8.92)	14.06	4.52	58.29 (10.88)	41.4
	Duloxetine	60 mg BID	3 days	116		5.65 (1.29)	85.3	13.93 (9.72)	11.35	4.47	58.98 (9.58)	52.6
	Placebo			116		5.47 (1.25)	87.9	12.83 (8.64)	11.32	3.98	59.2 (9.84)	45.7

***Pregabalin***												
DPN-029	Pregabalin	100 mg TID	1 wk	82	5	6.2 (1.4)	93.8		7		59 (9.2)	59.3
	Pregabalin	200 mg TID	1 wk	82		6.2 (1.5)	92.7		6.5		62 (9.7)	63.4
	Placebo		1 wk	97		6.6 (1.5)	85.6		8		57.8 (11.6)	60.8

DPN-131	Pregabalin	100 mg TID		76	8	6.5	84.2	9.4 (10.3)	6		59.2 (12.3)	55.3
	Placebo			70		6.1	90	9.3 (10.5)	5.5		60.3 (10.3)	57.1

DPN-014	Pregabalin	50 mg TID	2 wks	79	6	6.5	91.1		4		56.3 (9.4)	
	Pregabalin	200 mg TID	2 wks	82		6.7	97.6		6.5		57.8 (9.5)	
	Placebo		2 wks	85		6.9	83.5		9		57.1 (10.3)	

DPN-040	Pregabalin	200 mg TID	2 wks	87	8	6.9	87.2		10.5		62 (9.4)	
	Amitriptyline	25 mg TID	2 wks	88		6.4	82.8		9		57.8 (12.0)	
	Placebo		2 wks	81		6.3	87.7		11		60.6 (11.5)	

DPN-149	Pregabalin	75 mg BID	1 wks	99	12	6.2	86		12		58.5 (12.6)	
	Pregabalin	150 mg BID	1 wks	99		6.4	84		12.5		57.6 (10.5)	
	Pregabalin	150/300 mg BID	1 wk	101		6.6	86		11		59.5 (11.4)	
	Placebo		1 wk	97		6.4	86		11		58.8 (11.8)	

DPN-155	Pregabalin	600 mg QID	1 wk	96	8	6.67	82.3	13.2	11.4		61.8 (11)	54.5
			
	Pregabalin	Flexible dose 150 – 600 mg	1 wk	105		6.67	84.8	13.8	10.4		62.7 (10.6)	52.5
			
	Placebo			48		6.55	81.3	13.6	11.5		61.7 (12.6)	56.9

***Gabapentin***												
Backonja 1998	Gabapentin	1200 mg TID	4 wks	84	8	6.4	75	12 (9.6)			53 (10.5)	58.3
	Placebo		4 wks	81		6.5	75	11.2 (8.7)			53 (10.5)	61.7

Simpson 2001	Gabapentin	1200 mg TID	4 wks	30	8	6.4	80	8 (7.4)			48 (8.2)	60
	Placebo		4 wks	30		6.5	83	9 (7.8)			52 (9.8)	60

*Note: duloxetine trial arms with dose 20 mg QD and pregabalin trial arms with dose 25 mg TID were excluded from the meta-analysis;

^a^QD: Once daily; BID: Twice daily; TID: Three times daily.

### Meta-analysis

#### Direct comparison results

As heterogeneity between studies measured by the Q-test was not significant and the FE and RE models produced similar estimates of treatment effect for the primary and the other outcomes, only the results from the random-effects models are reported here.

All three drugs were superior to placebo for all efficacy parameters. (Please note: effects (*θ*) favouring drug over placebo take a negative sign for the outcome measures 24 h PS, PGI-I/C and tolerability but a positive sign for response). For 24 h PS, *θ *(95% CIs) were DLX: -1.13 (-1.36; -0.89), PGB: -0.90 (-1.23; -0.57), GBP: -1.44 (-2.21; -0.66). Corresponding *θ *values for response rate were DLX: 0.86 (0.63; 1.09) with NNT of 5 (3; 7), PGB: 0.84 (0.52; 1.16) with NNT of 5 (4; 8); and for PGI-I/C were DLX: -0.76 (-1.00; -0.51), PGB: -1.29 (-1.72; -0.86).

**Figure 2:**
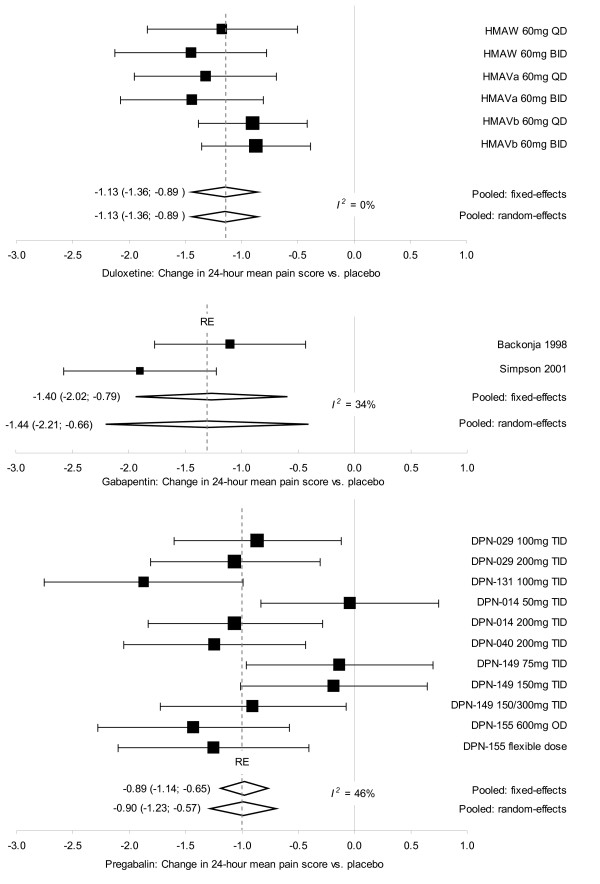
**Forest plots: change in average 24-hour pain score, direct comparisons with placebo**.

The forest plots in Figure 2 show point estimates and 95% CIs for the primary efficacy outcome, 24 h PS, for individual trials of each drug, along with pooled FE and RE estimates and I-squared statistics.

Table 2 presents the results of the RE analyses of DLX vs. placebo. DLX was statistically significantly more effective than placebo on all three efficacy variables. The U-test was statistically significant (indicating the existence of treatment effects) for all efficacy outcomes and all tolerability outcomes except discontinuation due to other reason and diarrhoea. DLX resulted in significantly lower premature discontinuation due to lack of efficacy than placebo. Premature discontinuation due to adverse events was significantly more common for DLX than for placebo (NNH = 11 (95% CI: 7; 23)). Between-study variance (*τ*^2 ^≠ 0) was detected for only two of the individual adverse event outcomes (diarrhoea and nausea). For the individual tolerability outcomes, DLX gave rise to a significantly higher incidence of dizziness, headache, nausea and somnolence than did placebo.

**Table 2:** Random-effects pooled results: duloxetine vs. placebo.

**Outcome**	**Treatment effect **(*θ*)	**95% CI for *θ***	**U-test** (p-value)	***τ*^2^**	***NNT/NNH*** *(95% CI)*
***Efficacy***					
					
Reduction in 24-hour pain intensity	-1.128	(-1.364; -0.891)	<0.001	0	-
Response	0.856	(0.628; 1.085)	<0.001	0	5(3;7)
PGI	-0.756	(-1.004; -0.508)	<0.001	0	-

***Tolerability***					
					
Premature study discontinuation due to:					
- Lack of efficacy	-0.962	(-1.800; -0.124)	(0.024)	0	
- Adverse events	1.077	(0.663; 1.490)	<0.001	0	*11 (7; 23)*
- Other	-0.278	(-0.636; 0.079)	(0.127)	0	
Diarrhoea	0.233	(-0.436; 0.903)	(0.307)	0.307	
Dizziness	0.817	(0.398; 1.235)	<0.001	0	
Headache	0.468	(0.090; 0.845)	(0.015)	0	
Nausea	1.306	(0.942; 1.669)	0.039	0.039	
Somnolence	1.472	(1.044; 1.900)	<0.001	0	

Note:

*θ *is absolute difference for 24-hour pain intensity.

*θ *is log-odds ratio for Response, PGI and all tolerability analyses.

*τ*^2 ^is between-study heterogeneity.

*NNT/NNH: *Number needed to treat, number needed to harm. NNTs were calculated for response rate and NNHs were calculated for discontinuation due to adverse events.

Table 3 presents the results of the RE analyses of PGB vs. placebo. As for DLX, PGB was significantly more effective than placebo on all three efficacy variables. A significantly lower rate of premature discontinuation due to lack of efficacy was seen for PGB than for placebo. Premature discontinuation due to AEs occurred significantly more frequently for PGB than for placebo (NNH = 19 (95% CI: 10; 48)). Heterogeneity between PGB studies was observed for all efficacy variables and for diarrhoea and dizziness. PGB gave rise to a significantly higher incidence of dizziness and somnolence than did placebo.

**Table 3:** Random-effects pooled results: pregabalin vs. placebo.

**Outcome**	**Treatment effect **(*θ*)	**95% CI for *θ***	**U-test** (p-value)	***τ*^2^**	***NNT/NNH*** *(95% CI)*
***Efficacy***					
					
Reduction in 24-hour pain intensity	-0.901	(-1.234; -0.568)	<0.001	0.147	
Response	0.840	(0. 524; 1.155)	<0.001	0.154	5(4;8)
PGI	-1.291	(-1.722; -0.860)	<0.001	0.019	

***Tolerability***					
					
Premature study discontinuation due to:					
- Lack of efficacy	0.713	(-1.205; -0.221)	(0.005)	0	
- Adverse events	0.926	(0.463; 1.389)	<0.001	0	*19 (10; 48)*
- Other	-0.209	(-0.721; 0.302)	(0.330)	0	
Diarrhoea	-0.660	(-1.734; 0.414)	0.139	0.139	
Dizziness	1.900	(1.314; 2.487)	0.028	0.028	
Headache	-0.216	(-0.823; 0.392)	0.486	0	
Somnolence	2.063	(1.361; 2.764)	<0.001	0	

Note:

*θ *is absolute difference for 24-hour pain intensity.

*θ *is log-odds ratio for Response, PGI and all tolerability analyses.

*τ*^2 ^is between-study heterogeneity.

*NNT/NNH: *Number needed to treat, number needed to harm. NNTs were calculated for response rate and NNHs were calculated for discontinuation due to adverse events.

Table 4 presents the results of the RE model for GBP vs. placebo. GBP was significantly superior to placebo for reduction in 24 h PS, the only efficacy outcome available in common for DLX and GBP. It was not possible to calculate an NNT for GBP, as binary responder rate data were unavailable. The incidence of dizziness and of somnolence was significantly greater for GBP than for placebo. For the remaining tolerability outcomes, no significant differences were found between GBP and placebo.

**Table 4:** Random-effects pooled results: gabapentin vs. placebo.

**Outcome**	**Treatment effect **(*θ*)	**95% CI for *θ***	**U-test** (p-value)	***τ*^2^**	***NNT/NNH*** *(95% CI)*
***Efficacy***					
					
Reduction in 24-hour pain intensity	-1.437	(-2.211; -0.663)	<0.001	0.109	*

***Tolerability***					
					
Premature study discontinuation due to:					
- Lack of efficacy	-1.066	(-2.786; 0.653)	0.224	0	
- Adverse events	0.241	(-0.786; 1.267)	0.646	0	*63 (30; ∞)*
- Other	-0.036	(-1.162; 1.090)	0.950	0	
Diarrhoea	0.393	(-0.555; 1.341)	0.416	0	
Dizziness	1.833	(0.834; 2.833)	<0.001	0	
Headache	1.146	(-0.018; 2.310)	0.054	0	
Nausea	0.595	(-0.532; 1.722)	0.301	0	
Somnolence	1.582	(0.643; 2.520)	0.001	0	

Note:

*θ *is absolute difference for 24-hour pain intensity.

*θ *is log-odds ratio for all tolerability analyses.

*τ*^2 ^is between-study heterogeneity.

*NNT/NNH: *Number needed to treat, number needed to harm. * NNT was not calculated, due to absence of required binary data on responder rates. NNH was calculated for discontinuation due to adverse events.

#### Indirect comparison results

The results of the indirect comparisons between DLX and PGB are presented in Table 5. For the primary efficacy outcome, reduction in 24 h PS, a difference of -0.248 (95% CI: -0.667; 0.162) was seen in favour of DLX. Hence, DLX was not inferior to PGB on this outcome, as the upper bound of the confidence interval did not exceed the non-inferiority margin of +2 points. For response, the difference between DLX and PGB was close to zero and not significant. For the patient global impression (PGI-I/PGI-C) outcomes, PGB showed an improvement of 0.542 points over DLX, a difference that just reached significance (95% CI: 0.016; 1.060). DLX produced a significantly lower incidence of dizziness than did PGB, with *δ *= -1.084 (-1.903; -0.317). In the other tolerability comparisons (premature discontinuation, diarrhoea, headache, somnolence), no statistically significant differences were found.

**Table 5:** Indirect comparison results: duloxetine vs. pregabalin.

**Outcome**	**Indirect treatment comparison **(*δ*) Mean (median)	**95% CI for *δ***	**Between-study variance **(*τ *^2^) Mean (median)	**95% CI for *τ*^2^**
***Efficacy***				
				
Reduction in 24-hour pain intensity	-0.248 (0.248)	(-0.667;0.162)	0.052 (0.024)	(0.001;0.252)
Response	0.033 (0.034)	(-0.393;0.451)	0.075 (0.052)	(0.001;0.287)
PGI-I/PGI-C	0.542 (0.545)	(0.016;1.060)	0.025 (0.009)	(0.001;0.151)

***Tolerability***				
				
Premature study discontinuation due to:				
- Lack of efficacy	-0.251 (-0.235)	(-1.288;0.717)	0.058 0.015)	(0.001;0.381)
- Adverse events	0.152 (0.154)	(-0.505;0.790)	0.039 (0.012)	(0.001;0.243)
- Other	-0.068 (-0.069)	(-0.735;0.589)	0.045 (0.013)	(0.001;0.281)
Diarrhoea	0.886 (0.885)	(-0.414; 2.183)	0.248 (0.050)	(0.001; 1.628)
Dizziness	-1.084 (-1.074)	(-1.903; -0.317)	0.075 (0.020)	(0.001; 0.477)
Headache	0.700 (0.704)	(-0.078; 1.458)	0.037 (0.011)	(0.001; 0.235)
Somnolence	-0.554 (-0.552)	(-1.458; 0.328)	0.052 (0.013)	(0.001; 0.347)

Note:

δ is the mean difference in treatment effect between DLX and each comparator.

*τ*^2 ^is between-study heterogeneity.

Table 6 presents the results from the indirect adjusted meta-analysis of DLX vs. GBP, in which no statistically significant differences were found.

**Table 6:** Indirect comparison results: duloxetine vs. gabapentin.

**Outcome**	**Indirect treatment comparison **(δ) Mean (median)	**95% CI for *δ***	**Between-study variance **(*τ *^2^) Mean (median)	**95% CI for *τ*^2^**
***Efficacy***				
				
Reduction in 24-hour pain intensity	0.270 (0.266)	(-0.469; 1.022)	0.041 (0.013)	(0.001; 0.247)

***Tolerability***				
				
Premature study discontinuation due to:				
- Lack of efficacy	0.067 (0.065)	(-1.988; 2.116)	0.177 (0.028)	(0.001; 1.281)
- Adverse events	0.841 (0.835)	(-0.348; 2.065)	0.062 (0.015)	(0.001; 0.406)
- Other	-0.245 (-0.252)	(-1.527; 1.075)	0.060 (0.015)	(0.001; 0.386)
Diarrhoea	-0.244 (-0.246)	(-1.645; 1.164)	0.273 (0.051)	(0.001; 1.825)
Dizziness	-1.044 (-1.054)	(-2.258; 0.183)	0.090 (0.021)	(0.001; 0.590)
Headache	-0.689 (-0.697)	(-1.986; 0.638)	0.053 (0.013)	(0.001; 0.348)
Nausea	0.704 (0.700)	(-0.567; 2.021)	0.085 (0.022)	(0.001; 0.529)
Somnolence	-0.101 (-0.107)	(-1.249; 1.078)	0.080 (0.016)	(0.001; 0.545)

Note:

δ is the mean difference in treatment effect between DLX and each comparator.

*τ*^2 ^is between-study heterogeneity.

## Discussion

This meta-analysis set out to compare the efficacy and tolerability of duloxetine, which is approved for the treatment of DPNP, with other drugs licensed or recommended in this indication. A variety of drugs, principally antidepressants, anticonvulsants and opioid analgesics, have been proposed for use in this difficult-to-treat condition. For example, controlled-release oxycodone has shown efficacy in diabetic painful neuropathy in two small trials [17,18]. However, at present the only formally licensed agents for DPNP are duloxetine, gabapentin and pregabalin, while amitriptyline is unlicensed but recommended for DPNP. The intended comparators to duloxetine in this study were, therefore, the tricyclic antidepressant amitriptyline and the γ-aminobutyric acid (GABA) analogues gabapentin and pregabalin.

Our approach to selecting efficacy outcomes for pooling was stricter than some previous meta-analyses in neuropathic pain. A common metric for 24 h PS based on an 11-point ordinal scale was used in the trials of duloxetine, gabapentin and pregabalin in DPNP. This allowed us to use this outcome directly and to construct from it a measure of responder rate (proportion of patients achieving 50% pain reduction). In reviews of unlicensed drugs and neuropathic pain more generally, the lack of standardisation of outcome measures across trials has required a looser definition of response rate. Previous reviews [19-21] have reported NNTs based on the criterion of 50% pain reduction, but NNTs have also been derived from various pain scales or approximations made where data were not available. For consistency, we have reported similarly-derived NNT (and NNH) values for the placebo comparisons.

DLX was statistically superior to placebo for all three efficacy outcomes and for premature discontinuation due to lack of efficacy. Duloxetine gave rise to significantly greater incidences of dizziness, headache, nausea, somnolence and premature discontinuation due to adverse events. A similar pattern was seen with PGB, which was statistically superior to placebo for the three efficacy outcomes and for premature discontinuation due to lack of efficacy. PGB produced a significantly greater incidence of somnolence, dizziness and premature discontinuation due to adverse events. GBP was significantly superior to placebo in reducing 24 h PS and produced significantly greater scores for dizziness and somnolence than did placebo.

Our estimated NNT of 5 (95% CI: 3; 7) for the pooled studies of DLX compares with the NNTs reported elsewhere of 5.2 (95% CI: 3.7; 8.5) [22] and 4.1 (95% CI: 2.9; 7.2) [20], the latter estimate based on the first published trial of DLX [23]. Our estimated NNT for PGB (5 (95% CI: 4; 8)) was slightly higher than a previously reported value (4.2 (95% CI: 3.4; 5.4)) [24], due to our inclusion of studies DPN-149 and DPN-040. The NNTs for the individual PGB studies were very similar to those provided in a recent review [25]. Data on GBP were limited and did not provide information on treatment response. Recent reviews reported NNTs of 3.4 (95% CI: 2.1; 5.4) or 3.8 (95% CI: 3.4; 5.4) in DPNP for GBP [24,26]. Otherwise, previous meta-analyses in neuropathic pain differ from the present study in indications and drugs studied, so are not directly comparable. Two reviews [20,26] concluded that across the neuropathic conditions relieved by these agents, the tricyclic antidepressants (TCAs) appear to have the lowest NNT, at approximately 3, though as noted in the recent EFNS guidelines [22] the individual trials are invariably small, crossover studies that may overestimate the efficacy of TCAs.

Indirect adjusted comparisons were performed between DLX and PGB and between DLX and GBP, using placebo as the common comparator. Comparing DLX with PGB, only two comparisons reached significance: PGI-I/PGI-C, which favoured PGB, and the incidence of dizziness, which favoured DLX. In the comparisons between DLX and GBP, no statistically significant differences were detected.

Meta-analysis can be a powerful tool for comparing treatments across individual clinical trials, but caution is needed in its application and in the interpretation of results. We comment below on some limitations of the present study. The precision of the effect size estimated by meta-analysis depends in part upon the number of contributing trials and their size. Only in the last few years have formal clinical programs to support regulatory approval of drugs proposed for use in DPNP been carried out. This is reflected in the small number of trials that met the eligibility criteria for this meta-analysis.

In particular, the exclusion of amitriptyline from the meta-analysis warrants discussion as, along with other TCAs, it has been used in painful diabetic neuropathy for approximately 30 years. Although strong consensus exists for the clinical value of the TCAs, the clinical evidence stems from a series of small trials mainly conducted in the 1980s and 1990s, which were not designed to meet current regulatory requirements. As a result, relatively few studies met the formal criteria for inclusion in this meta-analysis. Among 20 articles describing the use of AMT in neuropathic pain, four were clinical trials of AMT monotherapy but only one of these, in which AMT and PGB were compared to placebo, met our pre-specified eligibility criteria. In that study, AMT achieved a numerically greater response rate than PGB. Of the two studies by Max, [27,28] the earlier was a small crossover study with no washout period between AMT and placebo. The later study randomised subjects to one of two crossover comparisons: AMT vs. desipramine and fluoxetine vs. placebo. Twenty patients completed both comparisons but no direct comparison of AMT and placebo was reported. The studies by Max employed different pain measures from those used in the DLX, GBP and PGB studies. Moreover, benzotropine was used as an active placebo to mimic the anticholinergic side-effects of amitriptyline. This was designed to preserve blinding but precluded any tolerability comparison between AMT and no treatment.

Nevertheless, systematic reviews have reported NNT/NNH values for AMT and the TCAs as a class, based on various definitions of response. For example, Finnerup [20] reported a pooled NNT for the TCAs in peripheral pain of 2.3 (95% CI 2.1–2.7). McQuay[21] reported an NNT for AMT of 2.1 (95% CI 1.5–3.5), based on Max[28]. This was calculated from the 15 of 29 patients on AMT and 1 of 29 on placebo who reported "complete", "virtually complete" or "a lot of" relief. McQuay reported an NNT of 15.3 (95% CI 3.5–8) for fluoxetine, based on 22 of 46 patients on fluoxetine and 19 of 46 on placebo who achieved "complete", "a lot of" or "moderate" relief studied by Max [27]. The only trial involving AMT using the response rate criteria of our meta-analysis was the regulatory trial DPN-040 of pregabalin, which included AMT as an active control as well as placebo. The NNT for response for AMT compared to placebo in this study was 5, rather higher than reported elsewhere. Therefore, when comparing NNT values across studies, it should be recognised that these depend, *inter alia*, on the underlying definitions of response. The accepted convention in pain studies is to define the NNT as the number of patients needed to treat to obtain one patient with at least 50% pain relief, but obtaining this statistic may require some subjectivity. In our meta-analysis, the outcome of 24 h PS measured on an 11-point ordinal scale was available across all included trials, so responder rates based on at least 50% pain relief could be obtained in a consistent manner. In other studies, various categorical scales have been used and judgement is required as to which levels of response correspond to 50% or greater pain relief. While we acknowledge that inclusion of AMT would have been desirable in view of the consensus for its clinical value and its widespread use in DPNP, the above data constraints precluded that possibility for this particular study.

Although opioids were excluded from this meta-analysis, as none is licensed in painful diabetic neuropathy, oxycodone has been shown to be effective in this indication in two randomized, placebo-controlled trials [17,18]. European guidelines [22] report a combined NNT for these trials of 2.6 (95% CI 1.9–4.1), though they warn that the eligibility of prior opioid users may have exaggerated the response rate.

Only two small studies of GBP were eligible. The three studies of DLX and six studies of PGB involved more patients, reflecting that these were trials designed to meet regulatory authorities' requirements. Pooled estimates from small numbers of trials may gain little in precision over estimates from the individual trials. This is not necessarily a concern where trials are large and adequately powered, where differences in effect sizes are large, or both. However, the two studies of GBP contributed only 114 patients on active drug, and pooling them produced only modest shrinkage in the confidence intervals. Moreover, concerns have been expressed that small studies tend to suffer from design shortcomings, such that they over-estimate effect size [29]. It was not possible to calculate an NNT for GBP. Therefore, we believe that the most important and valid results in this study are the direct comparisons of DLX and PGB with placebo and the indirect comparisons between DLX and PGB. Investigating the validity of indirect adjusted comparisons, Song [30] found that the results of indirect comparisons usually, but not always, agree with results from head-to-head randomised clinical trials, and concluded that in the absence of direct evidence, adjusted indirect comparisons may provide useful information.

The possibility of study selection bias is a potential threat to the validity of meta-analyses. For DLX and PGB, we are confident that all studies in DPNP were identified, and that selection bias was therefore unlikely. As of the date the literature search was performed, the manufacturer provided study reports of all completed trials of DLX, none of which had been published. The three eligible trials of DLX have since been published [23,31,32]. No relevant trials have been conducted since the time of the search. We did not contact the manufacturer of PGB and GBP to identify unpublished studies, as we considered that all relevant evidence would have been disclosed to the FDA and EMEA, whose drug assessments are in the public domain. The European Public Assessment Report, which was published in December 2004, only one month prior to our literature search, stated that it included all six completed trials of PGB in DPNP. Two of these six trials had been published at the time of search[15,16], and a further three [33-35] had been published as of October 2008. The apparently unpublished study is DPN-040, which included AMT as an active comparator. All six studies we identified, along with a recently completed seventh trial [36] not revealed by our search, have recently been the subject of an article [37] reviewing dose response to PGB. For GBP, which is a patent-expired drug, it is unlikely that any recent (unpublished) manufacturer-sponsored trials have been conducted.

The systematic review process to determine study eligibility appears to have resulted in a reasonably homogeneous selection. Trial designs shared many common features, outcome metrics and patient characteristics (except for gender and proportion with type 2 diabetes), and we detected little evidence of overall heterogeneity between the trials included. With respect to the between-study variability in outcomes for each drug, there were no significant differences, but we conservatively chose to report RE as well as FE models in the drug vs. placebo comparisons.

To demonstrate the equivalence of two drugs, it is common practice to perform tests of non-inferiority. In the context of DPNP, 24 h PS is the only efficacy outcome for which a clinically meaningful non-inferiority margin has been documented, based on the work of Farrar [8], who proposed a margin of two points on the 11-point pain intensity rating scale. However, it was unnecessary to invoke this non-inferiority margin in the comparisons of DLX with PGB and GBP. Using the stricter criterion of equivalence, there was no difference in the 24 h PS scores between DLX and each of these comparator drugs, and we conclude that DLX is equivalent to GBP and PGB on this outcome. Although PGB showed a statistically greater score than DLX on the PGI-I/PGI-C outcome, it is not clear that the measured difference of approximately 0.5 points is clinically meaningful. This categorical seven-point scale describes "very much improved", "much improved", "minimally improved", "no change", "minimally worse", "much worse" and "very much worse". Intuitively, it is difficult to conclude that less than a whole interval change on this scale is meaningful. If a whole interval were accepted as the non-inferiority margin, DLX would be non-inferior to PGB in terms of patients' global impressions.

The indirect comparisons of tolerability were, by definition, limited to those outcomes for which data were reported in common for each comparator. One outcome for which a comparison was not possible in this study was weight gain. The European Public Assessment Report refers to dose-related weight gain with PGB (2.4–8.2% of patients), compared to 0.8% of placebo-treated patients, over all neuropathic pain indications. Among DPNP patients for whom data were available, the mean weight gain over the assessment period was 1.6 kg for PGB-treated patients and 0.3 kg for placebo-treated patients. In the Integrated Safety Summary for DLX in diabetic neuropathic pain, DLX did not differ significantly from routine care or placebo controls in the incidence of weight gain.

## Conclusion

Based on the pooling of common outcomes measured in randomized, controlled trials, we conclude that duloxetine is comparably effective and tolerable in the treatment of diabetic peripheral neuropathic pain to two anticonvulsants, gabapentin and pregabalin, which are pharmacologically unrelated to duloxetine. The summary reporting of trial results tends to conceal that response and tolerability to the various types of pharmacological treatment may be highly individual. In neuropathic pain, the empirical approach to treatment and the common use of off-label treatments attest to the clinical need for a wide range of drug choices. This study suggests that duloxetine may offer a valuable, additional option for this disabling condition.

## Competing interests

TKL and AG-C are employees of Eli Lilly and Company and BM is an employee of Boehringer Ingelheim. These two companies market duloxetine, which is indicated for DPNP, in the European Union. Eli Lilly and Company reimbursed i3 Innovus for payment of the Journal's article processing charge. JC is a current employee, and SQ is a former employee, of i3 Innovus, a business unit of Ingenix Pharmaceutical Services Limited, which carried out the study reported here under a commercial contract with Eli Lilly and Company. ML is a former employee of Innovus Research (UK) Ltd, which was acquired by Ingenix in 2006. Funding was not contingent upon publication of the manuscript. GS and DS have received fees as occasional advisors to Eli Lilly and Company and DS has received financial support from Lilly to conduct a pilot study on the determinants of diabetes-related complications. None of the authors hold stocks or shares in Eli Lilly and Company or Boehringer Ingelheim, the latter company being privately owned. No patents have been applied for relating to the content of the manuscript. None of the authors has any other financial or non-financial competing interests.

## Authors' contributions

The systematic review, the selection of included studies and outcome measures, analysis or reporting of results were performed entirely independently by those authors who are (JC) or were (SQ, ML) employees of i3 Innovus or Innovus Research (UK) Ltd. Protocols for the systematic review and meta-analyses were developed by i3 Innovus (ML), reviewed and commented on by all authors, and amendments were made based on the recommendations of Professors Simon and Said. The systematic review (ML), analyses (SQ) and manuscript preparation (JC) were performed by i3 Innovus; the manuscript was reviewed and agreed by all co-authors.

## Pre-publication history

The pre-publication history for this paper can be accessed here:


